# Clinical features and outcomes of infective endocarditis in persons experiencing homelessness

**DOI:** 10.1186/s40249-025-01318-4

**Published:** 2025-06-13

**Authors:** Torrance Teng, Kyle Crooker, Tess Hickey, Max HoddWells, Ashwini Sarathy, Sean Muniz, Jennifer Lor, Amy Chang, Bradley J. Tompkins, Aaron O’Brien, Elly Riser, Devika Singh, Jean Dejace, Andrew J. Hale

**Affiliations:** Infectious Disease Unit, University of Vermont Medical Center, 111 Colchester Avenue, Mailstop 115 SM2, Burlington, VT 05401 USA

**Keywords:** Endocarditis, Homelessness, Undomiciled, Underserved

## Abstract

**Background:**

Infective endocarditis (IE) is associated with significant morbidity and mortality and current treatment guidelines recommend a prolonged course of intravenous antibiotics. However, individuals experiencing homelessness face greater barriers to standard IE care. The aim of this study was to compare IE characteristics and outcomes in an unhoused population versus a housed population.

**Methods:**

A retrospective cohort study encompassing 2010–2020 was conducted in Burlington, Vermont, comparing characteristics and outcomes of patients with IE who did or did not experience homelessness at the time of their infection. Primary outcomes included 30-day, 90-day, and 365-day mortality, IE-related mortality, and IE-related readmission rates. Secondary outcomes included rates of microbiologic failure and treatment failure.

**Results:**

Of 378 included patients with IE, 30 (7.9%) experienced homelessness and 348 (92.1%) did not. The unhoused cohort was more likely to have right-sided IE involving the tricuspid valve (50.0% vs 21.6%, *P* = 0.006) and for the causative organism to be methicillin-resistant *Staphylococcus aureus* (MRSA) [9 (30.0%) vs 43 (12.4%), *P* = 0.039]. Mortality, IE-related mortality, and IE-related readmission rates were not significantly different between groups at any time point measured. For secondary outcomes, differences in microbiologic failure [5 (16.7%) vs 36 (10.3%), *P* = 0.543] and treatment failure [9 (30.0%) vs 105 (30.2%), *P* = 1.000] were also not statistically significant.

**Conclusions:**

Future research should elucidate factors that entail increased risk of poor outcomes in this important underserved population and how to best mitigate them.

## Background

Infective endocarditis (IE) is an infection of the endocardium that results in significant morbidity and mortality [1]. IE can cause substantial damage to the body, resulting in severe multi-organ system damage and death with a mortality rate approaching 30% at 30 days [2]. Standard treatment entails 4–6 weeks of intravenous antibiotics though similar efficacy has been shown for select patients with a transition to oral antibiotics after an initial two-week intravenous antibiotic phase [3–6]. However, up to 50% of cases require surgical intervention such as for patients with large vegetations or comorbidities like severe heart failure [3–5, 7]. Though adherence with a 4–6 week course of antibiotics in addition to collaborative care from a multi-personnel team has improved patient outcomes, individuals diagnosed with IE still face high morbidity and mortality rates [8, 9].

In recent years, the incidence of IE in the United States rose from 3.7 new cases per 1,000,000 persons per day in 2011 to 30.1 new cases per 1,000,000 persons per day in 2022, with a noted increased incidence rate starting in 2020 [10–12]. Furthermore, there has also recently been an observed increase in IE related mortality, specifically among adults between 25 and 44 years old [10–12]. This is likely related to the opioid crisis and an increased prevalence of substance use disorder [11]. People experiencing homelessness, of whom there are over 550,000 individuals daily or roughly 2.5–3.5 million people per year in the United States, have higher rates of injection drug use compared to the general population [13–15]. In addition, those experiencing homelessness face significant barriers to receiving the standard of care for IE including social stigma and socioeconomic disparities, which leads to increased healthcare complications and, often, worse healthcare outcomes [16, 17]. However, there is a paucity of data assessing the clinical features, treatments, and outcomes of IE in patients experiencing homelessness. One study from 2021 analyzed a national inpatient database and found an increased incidence of IE in patients experiencing homelessness from 2000 to 2017 [18]. The study was broad in its analysis but showed advanced age and alcohol use disorder was independently associated with mortality in the unhoused population. However, though there was a trend towards increased mortality in the unhoused cohort with noted lower utilization of valvular surgeries possibly secondary to barriers to proper healthcare access, there was no observed significant difference in mortality of the unhoused group compared to the general population [18]. Since that study was conducted, the incidence of infective endocarditis in patients experiencing homelessness has only risen, recently been estimated to be almost 40 times the incidence of the general population and worsened during the COVID-19 pandemic [15, 18, 19]. It is thus essential for an updated and thorough analysis of the clinical features and outcomes of infective endocarditis in this underserved population.

The goals of this study are to investigate demographics, clinical features, and short- and long-term outcomes in patients with IE experiencing homelessness, compared to those who do not. We hypothesized that patients experiencing homelessness encounter greater barriers to treatment and suffer worse outcomes.

## Methods

### Study design, setting, population, and ethical approval

We conducted a retrospective cohort analysis comparing demographics, clinical features, and outcomes of patients with infective endocarditis (IE) who did or did not experience homelessness at the time of presentation. Patients experiencing homelessness were identified via chart review. The study consisted of adult patients admitted from 2010 to 2020 at the University of Vermont Medical Center, a tertiary care academic hospital, with 620 licensed beds in Burlington, Vermont that serves an urban and rural population of about 1 million people. Patients were identified via ICD-10 codes (I33.0 and I33.9). Inclusion criteria were age greater than 18 years and a diagnosis of IE as determined by the modified Duke criteria [20]. Exclusion criteria included not meeting an IE diagnosis per modified Duke criteria, even if an IE ICD-10 code was used. Each chart was reviewed by two study team members and any discrepancies were resolved by a third physician review. This study was approved by the UVM Institutional Review Board.

### Outcomes

Primary outcomes included 30-day, 90-day, and 365-day mortality, IE-related mortality, and IE-related readmission rates. Secondary outcomes included rates of microbiologic failure (recurrence of bacteremia with the original organism after completion of planned treatment) and treatment failure (death due to endocarditis as determined by reviewer, new paravalvular abscess, new heart failure from IE, new need for valve replacement, new need for an IE related orthopedic procedure, or a new metastatic site of infection).

### Statistical analysis

Study data was collected into a REDCap electronic data capture tool hosted at The University of Vermont [21, 22]. Stata (Stata 16.1, Stata Corp, LLC. College Station, TX) was used for statistical analysis. We compared demographics, clinical findings, and outcomes between patients with IE who did or did not experience homelessness via Wilcoxon rank sum test for continuous variables and via chi-square analysis or Fisher’s exact test for categorical variables. The* P*-values were corrected for multiple comparisons via false discovery rate (FDR) method [23]. Adjusted P-values are listed in manuscript unless stated otherwise.

## Results

### Demographics

There were 378 adult infective endocarditis patients eligible for inclusion and analysis in this study. Of this group, 30 (7.9%) identified as experiencing homelessness at the time of IE diagnosis and 348 (92.1%) did not. Demographic data is shown in Table 1. The cohort of patients experiencing homelessness was significantly younger (median age of 35.9 years old vs 59.8 years old, *P* = 0.006) and more likely to be insured with Medicaid than with other types of insurances [21 (70.0%) vs. 107 (30.8%), *P* = 0.006]. Additionally, they were more likely to have substance use disorders including smoking tobacco [24 (80.0%) vs 124 (36.9%), *P* = 0.006] and active intravenous drug use [26 (86.7%) vs 104 (29.9%), *P* = 0.006]. However, they had fewer chronic comorbidities such as chronic heart failure [0 (0.0%) vs 54 (15.5%),* P* = 0.061] or diabetes [2 (6.7%) vs 81 (23.3%), *P* = 0.144], though they were both not significant after adjusting *P*-values via FDR method.

**Table 1:** Comparison of baseline demographics between patients who were or were not experiencing homelessness at the time of infective endocarditis diagnosis

	Not experiencing homelessness (*n* = 348) (%)		Experiencing homelessness (*n* = 30) (%)		Adjusted *P-*value/*P*-value
Age (years) (interquartile range)	59.8	(36.2–72.4)	35.9	(31.6–44.3)	0.006 < 0.001
Gender: female	139	(39.9)	11	(36.7)	1.000 0.725
Race: Caucasian	331	(96.8)	28	(93.3)	0.543 0.282
Ethnicity: non-Hispanic	335	(99.1)	30	(100.0)	1.000
Insurance: Medicaid	107	(30.8)	21	(70.0)	0.006 < 0.001
Tobacco use disorder	124	(36.9)	24	(80.0)	0.006 < 0.001
Alcohol use disorder	52	(15.1)	8	(27.6)	0.224 0.078
Current injection drug use	104	(29.9)	26	(86.7)	0.006 < 0.001
Other current substance use	104	(30.1)	21	(70.0)	0.006 < 0.001
Methamphetamines	16	(4.6)	4	(13.3)	0.204 0.065
Cocaine	55	(15.9)	14	(46.7)	0.006 < 0.001
Opioids	49	(14.2)	9	(30.0)	0.091 0.022
Chronic kidney disease	74	(21.3)	2	(6.7)	0.195 0.059
Cirrhosis	21	(6.0)	1	(3.3)	1.000
HIV infection	3	(1.1)	0	(0.0)	1.000
HBV infection	3	(1.1)	1	(3.3)	0.627 0.342
HCV infection	81	(28.4)	22	(73.3)	0.006 < 0.001
History of psychiatric disease	123	(35.3)	13	(43.3)	0.681 0.382
Pre-existing prosthetic heart valve	72	(20.7)	2	(6.7)	0.238 0.090
Indwelling cardiac device	40	(11.5)	0	(0.0)	0.195 0.058
Coronary artery disease	81	(23.3)	3	(10.0)	0.279 0.111
Chronic heart failure	54	(15.5)	0	(0.0)	0.061 0.013
Diabetes	81	(23.3)	2	(6.7)	0.144 0.037
Immunocompromised	14	(4.0)	0	(0.0)	0.881 0.614
Prior to admission, on methadone (% of total cohort with intravenous drug use)	20	(19.2)	7	(26.9)	0.006 < 0.001
Prior to admission, on buprenorphine/naloxone (% of total cohort with intravenous drug use)	54	(51.9)	8	(30.8)	0.279 0.114

### Clinical features

Clinical features between these two groups are shown in Table 2. In the persons experiencing homelessness cohort, there was a significantly higher rate of patient-directed discharges [8 (26.7%) vs 37 (10.6%), *P* = 0.046] and behavioral concerns during hospitalization [9 (30.0%) vs 29 (8.3%), *P* = 0.006], the latter of which included room searches, security involvement, and suspected drug use per hospital staff documentation. However, the mean length of hospitalization [27.9 days vs 20.2 days, *P* = 0.283] and mean IV antibiotic duration [34.5 days vs 34.0 days, *P* = 0.786] between the two groups were not significantly different. The persons experiencing homelessness group was more likely to have right-sided IE involving the tricuspid valve [15 (50.0%) vs 75 (21.6%),* P* = 0.006] and the housed cohort was more likely to have left-sided IE involving the aortic valve [103 (29.6%) vs 3 (10.0%), *P* = 0.088], though the latter was not significant after adjusting p-value via FDR; unadjusted *P* = 0.020. The unhoused cohort was also noted to have the causative organism be Methicillin-resistant *Staphylococcus aureus* (MRSA) [9 (30.0%) vs 43 (12.4%),* P* = 0.039]. The patients experiencing homelessness group were also more likely to have metastatic infection to the lung [18 (60.0%) vs 73 (21.0%), *P* = 0.006] while patients not experiencing homelessness were more likely to have metastatic infection to the brain [4 (13.3%) vs 75 (21.6%), *P* = 0.543], though the latter was not statistically significant.

**Table 2:** Clinical characteristics of infective endocarditis episodes in patients experiencing homelessness compared to patients not experiencing homelessness

	Not experiencing homelessness (*n* = 348) (%)		Experiencing homelessness (*n* = 30) (%)		Adjusted *P-*value/*P*-value
Pitt Bacteremia score (IQR)	1	(0–2)	1	(0–1)	0.881 0.599
Behavioral concern during admission	29	(8.3)	9	(30.0)	0.006 < 0.001
Heart valves infected					
Tricuspid	75	(21.6)	15	(50.0)	0.006 < 0.001
Pulmonic	3	(0.9)	1	(3.3)	0.543 0.283
Aortic	103	(29.6)	3	(10.0)	0.088 0.020
Mitral	101	(29.0)	5	(16.7)	0.447 0.203
Unknown	51	(14.7)	6	(20.0)	0.713 0.432
Paravalvular abscess	11	(3.2)	1	(3.3)	1.000
New heart failure from IE	61	(17.5)	4	(13.3)	1.000 0.801
Microbiology					
MSSA	115	(33.1)	13	(43.3)	0.539 0.253
MRSA	43	(12.4)	9	(30.0)	0.039 0.007
*Strep viridans*	31	(8.9)	1	(3.3)	0.781 0.494
Other Strep	62	(17.8)	1	(3.3)	0.160 0.041
*Enterococcus faecalis*	21	(6.0)	3	(10.0)	0.713 0.423
*Enterococcus faecium*	1	(0.3)	0	(0.0)	1.000
HACEK group	7	(2.0)	0	(0.0)	1.000
*Candida*	4	(1.2)	0	(0.0)	1.000
Sites of metastatic infection					
Lung	73	(21.0)	18	(60.0)	0.006 < 0.001
Brain	75	(21.6)	4	(13.3)	0.543 0.288
Spine	16	(4.6)	3	(10.0)	0.419 0.184
Joint	34	(9.8)	2	(6.7)	1.000 0.755
Length of hospitalization – mean (IQR)	20.2	(17.2)	27.9	(22.1)	0.283 0.120
Length of hospitalization – median (IQR)	14	(7–28.5)	24.5	(7–44)	0.204 0.068
Days of positive blood cultures, median (IQR)	2	(1–3)	2	(1–4)	0.781 0.497
IV antibiotic duration in days, mean (SD)	34	(16.7)	34.5	(20.4)	0.786 0.512
Valve replacement surgery	69	(19.8)	2	(6.7)	0.238 0.089
Pacemaker placed	14	(4.0)	0	(0.0)	0.881 0.614
Orthopedic procedure	29	(8.3)	2	(6.7)	1.000
Number of patient directed discharges	37	(10.6)	8	(26.7)	0.046 0.009

*IE* infective endocarditis, *MSSA*  methicillin-sensitive *Staphylococcus aureus*, *MRSA* methicillin-resistant *Staphylococcus aureus*

### Outcomes

The primary and secondary outcomes can be found in Table 3. Mortality and IE-related mortality were not significantly different between the two cohorts at any time point measured. Differences in 30-day mortality rate [0 (0.0%) vs 11 (3.2%), *P* = 1.000], 90-day mortality rate [1 (3.3%) vs 19 (5.5%), *P* = 1.000], and 365-day mortality rate [10 (30.3%) vs 23 (7.8%), *P* = 0.681] were all not statistically different. IE-related mortality at 30-days [0 (0.0%) vs 9 (2.6%), *P* = 1.000], 90-days [0 (0.0%) vs 10 (2.9%), *P* = 1.000], and 365-days [0 (0.0%) vs 10 (3.4%), *P* = 1.000] were also not statistically significant. There was also no significant difference in readmission rates at 30-days [4 (13.3%) vs 47 (13.5%), *P* = 1.000], 90-days [6 (20.0%) vs 65 (18.7%), *P* = 1.000], and 365-days [6 (26.1%) vs 78 (26.5%), *P* = 1.000]. For secondary outcomes, differences in microbiologic failure [5 (16.7%) vs 36 (10.3%), *P* = 0.543] and treatment failure [9 (30.0%) vs 105 (30.2%), *P* = 1.000] were also not statistically significant.

**Table 3:** Outcomes of infective endocarditis episodes in patients who did or did not experience homelessness at the time of diagnosis

	Did not experienced homelessness (*n* = 348) (%)		Experienced homelessness (*n* = 30) (%)		Adjusted *P-*value/*P*-value
30-day mortality rate	11	(3.2)	0	(0.0)	1.000
90-day mortality rate	19	(5.5)	1	(3.3)	1.000
365-day mortality rate	23	(7.8)	10	(30.3)	0.681 0.392
30-day endocarditis related mortality rate	9	(2.6)	0	(0.0)	1.000
90-day endocarditis related mortality rate	10	(2.9)	0	(0.0)	1.000
365-day endocarditis related mortality rate	10	(3.4)	0	(0.0)	1.000
30-day readmission rate	47	(13.5)	4	(13.3)	1.000
90-day readmission rate	65	(18.7)	6	(20.0)	1.000 0.859
365-day readmission rate	78	(26.5)	6	(26.1)	1.000 0.963
Microbiologic failure	36	(10.3)	5	(16.7)	0.543 0.285
Treatment failure	105	(30.2)	9	(30.0)	1.000 0.984

## Discussion

This study helps clarify demographic, clinical, and outcome differences between patients with IE who do or do not experience homelessness. We demonstrated that the cohort experiencing homelessness was more likely to be younger, on Medicaid rather than other insurances, and have substance use disorder with active intravenous drug use leading to more right-sided endocarditis. Contrary to our hypothesis, overall mortality, IE-related mortality, and readmission rates between the two cohorts did not differ at 30, 90, or 365 days, indicating that the population experiencing homelessness was not at statistically increased risk for worse outcomes or complications. Additionally, this cohort did not have a statistically significant difference in length of hospitalization or length of antibiotic therapy. However, they had increased behavioral concerns such as room searches, concerns raised by the medical team for drug use or inappropriate behavior, security called for concerning behavior, and rates of patient-directed discharges during hospitalization. Nevertheless, they were not at an increased risk for complications like microbiologic failure or treatment failure.

This study is important as it is one of the few studies looking at endocarditis outcomes in people experiencing homelessness, a vulnerable population that has only been increasing in size and medical complexity in recent years [17, 18]. Our study is also one of the first to compare an IE population experiencing homelessness with one who is not, helping to elucidate potential differences. As has been seen in other studies, our patients experiencing homelessness were more likely to have a history of mental health disorders, substance use disorder, and active injection drug use which confer increased risk for acquiring IE and likely contributes to the increased incidence of infective endocarditis related hospitalizations in recent years [12, 15, 18]. This is supported by the 7.9% proportion of unhoused individuals in our IE cohort, which is higher than the previously noted nationwide proportion of 2.4% in 2017 [18]. Surprisingly, our study demonstrates the population experiencing homelessness was not at an increased risk for mortality despite these risk factors, similar to the findings documented by Khan et al. [18]. We theorize this to be secondary to the average younger age of the population experiencing homelessness, resulting in less concurrent age-related chronic diseases such as cardiovascular disease or diabetes mellitus. In addition, increased injection use in this population is more likely to lead to right-sided infective endocarditis which is inherently less dangerous than left-sided disease [24, 25].

This highlights that although our cohort experiencing homelessness was more likely to have risk factors for acquiring IE, they also tended to have less risky IE compared to the cohort without homelessness [26]. This amalgamation of factors for and against worse outcomes seems to have been balanced out such that we saw no significant differences in primary or secondary outcomes. Though there has been a limited number of studies addressing similar IE related outcomes in patients experiencing homelessness, the previous published study with related objectives demonstrated similar findings [18]. Although our study did not find statistically significant differences in mortality based on housing status, other significant differences were observed between groups including age, substance use, insurance status, and underlying co-infections such hepatitis C. Further research such as comparing follow-up rates among those with injection use in the unhoused and housed population provides one example that may help explore the impact of these variables on IE-related mortality and provide valuable insights. This is especially true in this geographical population since age and substance use were also independently associated with mortality among unhoused IE patients in the prior national study [18].

Patients experiencing homelessness remain an at-risk group, highlighting the need for further research to improve their overall health outcomes. It is well documented that the overall outcomes and mortality of people experiencing homelessness are worse than that of a housed population, with an average lifespan of about 17.5 years less than the general population [27]. Although they would benefit from further research aimed at improving morbidity and mortality outcomes, individuals experiencing homelessness continue to face negative biases from both healthcare providers and the public that negatively impact their ability to receive proper care [16, 17]. In our study, the people experiencing homelessness cohort had a statistically higher rate of behavioral concerns and patient-directed discharges which may be influenced by previous negative encounters with health care personnel related to their socioeconomic status and associated stigma that has limited their desire to seek medical help [16, 17, 28]. Nevertheless, our study showed that differences between rates of length of hospitalization and mean length of antibiotic therapy were not statistically significant. This was likely also influential in the lack of statistical differences for primary and secondary outcomes between the two groups in addition to the increased prevalence of right sided IE seen in the unhoused cohort. This data reflects the necessity of comprehensive and unprejudiced medical care to this marginalized population to minimize preventable adverse outcomes.

## Limitations

This study has several limitations. It was a retrospective study done on a relatively homogenous population at a single center, which may limit generalizability. The demographics of the studied population predominantly comprised of Caucasian, non-Hispanic individuals, limiting the generalizability of the finding. Although the sample size of the cohort experiencing homelessness was relatively small, it was sufficient to show statistically significant differences between the cohorts for many outcomes. Additionally, an extensive chart review was performed by multiple individuals, and this process was susceptible to recording errors during analysis. However, efforts were made to mitigate this risk through a cross-checking process. Patients may have been lost to follow-up, and their outcomes may not have been captured, which could undermine the conclusion that IE outcomes were similar between unhoused and housed individuals in our studied population. Lastly, to comprehensively characterize the study population, many variables were analyzed and compared. However, this could introduce bias and increase the possibility of Type I errors, despite our attempts to mitigate this by using the FDR method. This would be better assessed in future research utilizing a logistic regression model to independently account for other confounding variables including but not limited to age, sex, insurance status, substance use, and underlying co-infections.

## Conclusions

Though both the number of people experiencing homelessness and the rate of people experiencing homelessness with infective endocarditis has increased, our study suggests that patients with IE who are experiencing homelessness do not have a greater risk of mortality or readmission compared to those who are not. Future work in this field should elucidate factors that entail increased risk of poor outcomes in this important population and how best to mitigate them. This includes subpopulation analysis related to disparities in care including elements related to addiction, injection drug use, psychiatric care, follow-up care, and nature of antimicrobial therapy such as outpatient parenteral antimicrobial therapy.

## Data Availability

Data is available upon reasonable request.
